# The Middlesbrough Epidemic

**Published:** 1898-04-30

**Authors:** 


### THE MIDDLESBROUGH EPIDEMIC.

The Medical Officer of Health for Middlesbrough attributes the origin of the recent small-pox epidemic in that town to importation of infection from Bilbao, with which port Middlesbrough has a large trade, two or three vessels a day arriving all the year round from Bilbao and the neighbouring ports laden with ore. Small-pox has been very prevalent in that part of Spain lately, there having been no less than 418 deaths from the disease during the past year in Bilbao alone. The sudden diffusion of the disease, however, is looked upon as having been the result of a wake which was attended by "dozens of people." Of the 1,200 cases which had occurred up to the end of March, 1,078 cases were treated in hospital, those which were not removed having mostly occurred during the middle period of the epidemic, when its proportions entirely outran the accommodation which existed. Now there is provision for 822 beds, the largest number occupied at one time being 578. Our latest information gives the total number of cases as 1,331, and the deaths as 188.

